# Inherent High Correlation of Individual Motility Enhances Population Dispersal in a Heterotrophic, Planktonic Protist

**DOI:** 10.1371/journal.pcbi.1000942

**Published:** 2010-10-21

**Authors:** Susanne Menden-Deuer

**Affiliations:** University of Rhode Island, Graduate School of Oceanography, Narragansett, Rhode Island, United States of America; Princeton University, United States of America

## Abstract

Quantitative linkages between individual organism movements and the resulting population distributions are fundamental to understanding a wide range of ecological processes, including rates of reproduction, consumption, and mortality, as well as the spread of diseases and invasions. Typically, quantitative data are collected on either movement behaviors or population distributions, rarely both. This study combines empirical observations and model simulations to gain a mechanistic understanding and predictive ability of the linkages between both individual movement behaviors and population distributions of a single-celled planktonic herbivore. In the laboratory, microscopic 3D movements and macroscopic population distributions were simultaneously quantified in a 1L tank, using automated video- and image-analysis routines. The vertical velocity component of cell movements was extracted from the empirical data and used to motivate a series of correlated random walk models that predicted population distributions. Validation of the model predictions with empirical data was essential to distinguish amongst a number of theoretically plausible model formulations. All model predictions captured the essence of the population redistribution (mean upward drift) but only models assuming long correlation times (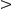
minute), captured the variance in population distribution. Models assuming correlation times of 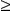
8 minutes predicted the least deviation from the empirical observations. Autocorrelation analysis of the empirical data failed to identify a de-correlation time in the up to 30-second-long swimming trajectories. These minute-scale estimates are considerably greater than previous estimates of second-scale correlation times. Considerable cell-to-cell variation and behavioral heterogeneity were critical to these results. Strongly correlated random walkers were predicted to have significantly greater dispersal distances and more rapid encounters with remote targets (e.g. resource patches, predators) than weakly correlated random walkers. The tendency to disperse rapidly in the absence of aggregative stimuli has important ramifications for the ecology and biogeography of planktonic organisms that perform this kind of random walk.

## Author Summary

Organism movement is fundamental to how organisms interact with each other and the environment. Such movements are also important on the population level and determine the spread of disease and invasion, reproduction, consumption, and mortality. Theoretical ecologists have sought to predict population dispersal rates, which are often hard to measure, from individual movement behaviors, which are often easier to measure. This problem has been non-trivial. This manuscript contributes seldom available, simultaneously measured movement behaviors and population distributions of a single celled planktonic organism. The empirical data are used to distinguish amongst a set of plausible theoretical modeling approaches to suggest that organism movements are highly correlated, meaning movement direction and speed is consistent over several minutes. Previous estimates suggested persistence only lasted several seconds. Minute-scale correlations result in much more rapid organism dispersal and greater dispersal distance, indicating that organisms encounter and impact a greater portion of their surrounding habitat than previously suspected.

## Introduction

Movement is fundamental to many ecological processes and often dictates relevant biotic and abiotic encounter rates, particularly for planktonic organisms inhabiting a highly dynamic and heterogeneous habitat. On the individual level, movement impacts encounter rates with favorable (e.g. mates, resources) and unfavorable (e.g. disease, consumers) targets. On the population level, these microscopic encounters directly affect growth and mortality rates, dispersal rates, population distributions, the spread of disease and invasion, home ranges, reproduction and survival (e.g. [1]). Particularly for micoroganisms, recent methodological advances have enabled the high resolution quantification of organism movements (e.g. [2]), their statistical features (e.g. [3]) and changes therein in response to external stimuli (e.g. [4]). Significant efforts have sought to establish mechanistic linkages between these individual movement behaviors and the resulting population distributions (reviewed in [5].) Deciphering these linkages for planktonic organisms, but also others, provides powerful tools to predict rates of organism encounters with environmentally relevant factors and ultimately, their ecological function.

Efforts to bridge the gap between individual movement behaviors and large scale population dispersal have been intense, especially in spatial ecology. Random walk theory has been a particularly powerful approach. Founded on observations of the irregular motions of pollen, i.e. Brownian motion [6], random walk theory relates organism movements in terms of speed, direction or turning rate to probabilities of particle distribution [7]–[9]. Correlated random walk models that assume correlation in successive movement direction, turning angle or velocity, have been particularly successful in linking movements and dispersion in diverse organisms [5], [10]–[12]. Every formulation of a random walk model rests on a set of assumptions about the underlying movement parameters, their changes over time and dependence on internal or external stimuli [13]. Predicted rates of population distributions are extremely sensitive to the underlying assumptions and to the exact model formulation [14]–[16]. As was recently shown for movement data of single-celled algae, widely used models with differing assumptions may yield significantly different predictions of organism distributions [17]. Thus, it is impossible to determine the most appropriate set of assumptions 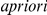 based on theoretical considerations alone.

Concurrent empirical data of both organism movements and their resulting population distributions and the stimuli that modulate these distributions are necessary to inform predictive model formulations. In a recent advancement, [18] have developed empirical methods that allow the simultaneous quantification of individual movement behaviors and population distributions of free swimming, planktonic organisms in stable and spatially structured environments. The approach taken here was to use these methods and empirically motivate a series of individual based, hidden Markov models to predict population distributions and examine the goodness of fit between model predicted and empirically measured distributions. The goals of this study were to (1) examine the feasibility of reproducing empirically observed population distributions from individual movement behaviors and (2) to identify the key characteristics necessary to adequately link individual movements with population distributions. Advancement on these goals is necessary to developing analytical solutions to random walks and predicting individual encounter rates, population distributions and ultimately the role of movement in driving organism abundance and distribution patterns. The results of both empirical and numerical analyses strongly suggest that motility patterns of some planktonic protists must have correlation times on the order of minutes.

## Results

**Figure 1:**
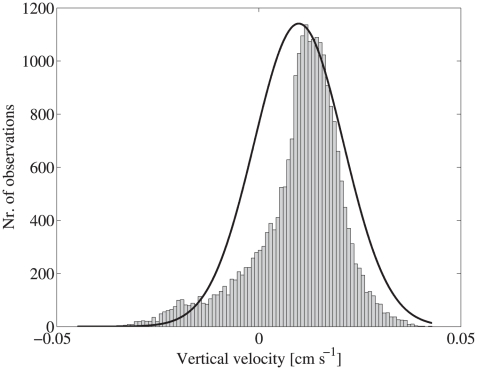
Frequency distribution of empirically measured vertical velocities for all swimming trajectories. Negative values indicate downward and positive values upward swimming. The probability density function of a normal distribution, with the same mean and variance, is superimposed to show the negative skew in the empirical data. This indicates that the empirical velocity data contained more and stronger downward swimmers and more, relatively weaker upward swimmers than normally distributed data.

Organism swimming behaviors and vertical distributions were measured in 3D using vertically moveable, stereo video cameras that recorded in randomized order at 6 vertically separate horizons. Each video segment yielded both individual movement behaviors and abundance of organisms. The footage was processed through a series of automated video-analysis steps that yielded organism positions, which were then used to reconstruct and analyze 3D movement behaviors. The empirical movement data consist of a total 1032 movement trajectories of *Oxyrrhis marina* swimming freely within 1L, 30 cm high column of filtered seawater, several cm distance from the nearest wall. The minimum trajectory length was 3 seconds, with 124 trajectories exceeding 10 seconds in duration. In total, these observations represent 108 minutes of movement data, with the median trajectories length 5.2 seconds and the longest observation 33 seconds. The mean swimming speed was 235 (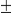
103) 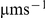 and the mean swimming direction was 57 (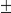
34) off the vertical axis. The frequency distribution of the over 24000 empirically determined vertical velocities shows that their distribution is non-gaussian, with a significant negative skewness (Fig. 1). Thus, the population was characterized by few strong down-swimmers and many, relatively slower up-swimmers. The median vertical velocity was 118 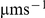 with a considerable standard deviation of 110 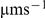. There was some indication that the population either underwent behavioral shifts during the time of observation, or that there were multiple behavioral types represented within this clonal lineage of *O. marina*. Vertical velocity significantly increased over the period of observation (p = 0.01), whereas there were no significant differences among vertical velocities measured at the six depths in the water column (p = 0.13). The frequency distribution of vertical velocities remained positively biased, irrespective of the time elapsed since introduction. Consistent upward swimming bias indicates that this bias was inherent to the organisms and not a function of the point of introduction at the base of the water column.

**Figure 2:**
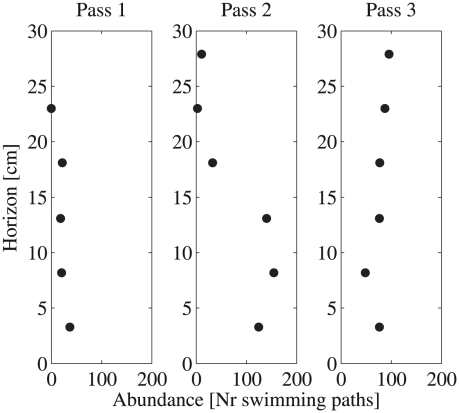
Empirical abundance of organisms observed at 6 vertical horizons in three successive passes, lasting 20–30 minutes each. Standard error of the abundance estimates are contained within the data symbols. The population was slowly moving upward and dispersed throughout the water column.

Simultaneously to measuring individual movement behaviors, the population distribution of *Oxyrrhis marina* was quantified throughout the entirety of the tank over 1.5 hours (Fig. 2). The time course of abundance changes are shown in three successive vertical profiles (i.e. passes) that each lasted 20–30 minutes. In the laboratory, the population showed a progressive upward drift, slowly increasing the number of cells at higher horizons. Because cells were introduced at the bottom of the tank, abundances at the upper horizons were initially low. Few individuals were seen rising upward rapidly, arriving at the top of the tank within the first 40 minutes (pass 1 and 2). The majority of individuals remained in the lower half of the tank for the first 40 minutes. After approximately 1 hr, the population appeared uniformly distributed throughout the tank.

**Figure 3:**
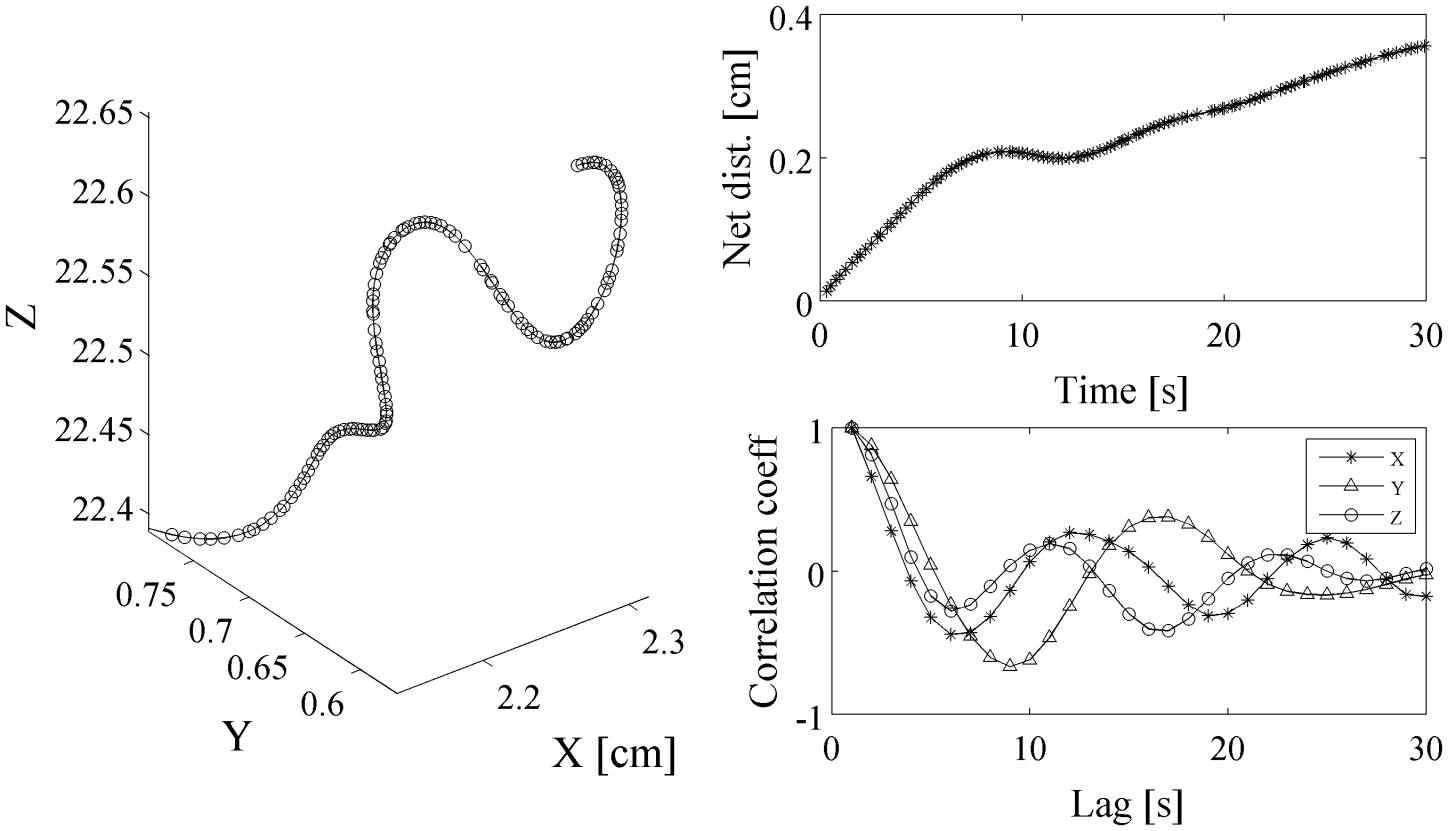
Empirical, 30-second, 3D-trajectory of *Oxyrrhis marina* (left panel) and corresponding net distance traveled (top, right panel) and autocorrelation coefficient for the three velocity components respectively (bottom, right panel). There was no evidence of a de-correlation in motion (i.e. transition from ballistic to diffusive motion). De-correlation would be indicated by a change in the slope of the line showing maximum distance travelled over time or by the correlation coefficients approaching zero. The cell continued to progress with a high degree of correlation even over 30 seconds. De-correlation was not observable in any of the paths recorded.

An individual-based, biased random walk model was formulated to establish linkages between individual movement behaviors on the microscopic level and the macroscopic population distributions and changes therein. To seed this model, individual movement behaviors needed to be characterized both in terms of vertical velocities as well as their correlation times. The movement paths showed highly periodic movements (Fig. 3, left panel), with correlation coefficients failing to asymptotically approach zero (right, bottom panel) and net distance traveled growing rapidly (left, top panel) as would be expected for highly correlated movements. Individual movement paths were characterized by high degrees of auto-correlation, in all three dimensions. De-correlation of velocities was not observable over the measured path durations. The auto-correlation coefficient calculated for the entirety of all trajectories failed to identify a de-correlation time in the up to 33 second long observations. However, sample size for trajectories 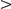
15 seconds was low, (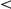
30 trajectories). Thus, autocorrelation analysis suggested that correlation times were 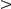
30 seconds but did not identify a distinct correlation time scale. Given this uncertainly, a range of 12 correlation times between 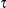 = 1 to 1800 seconds were chosen for the model analysis.

**Figure 4:**
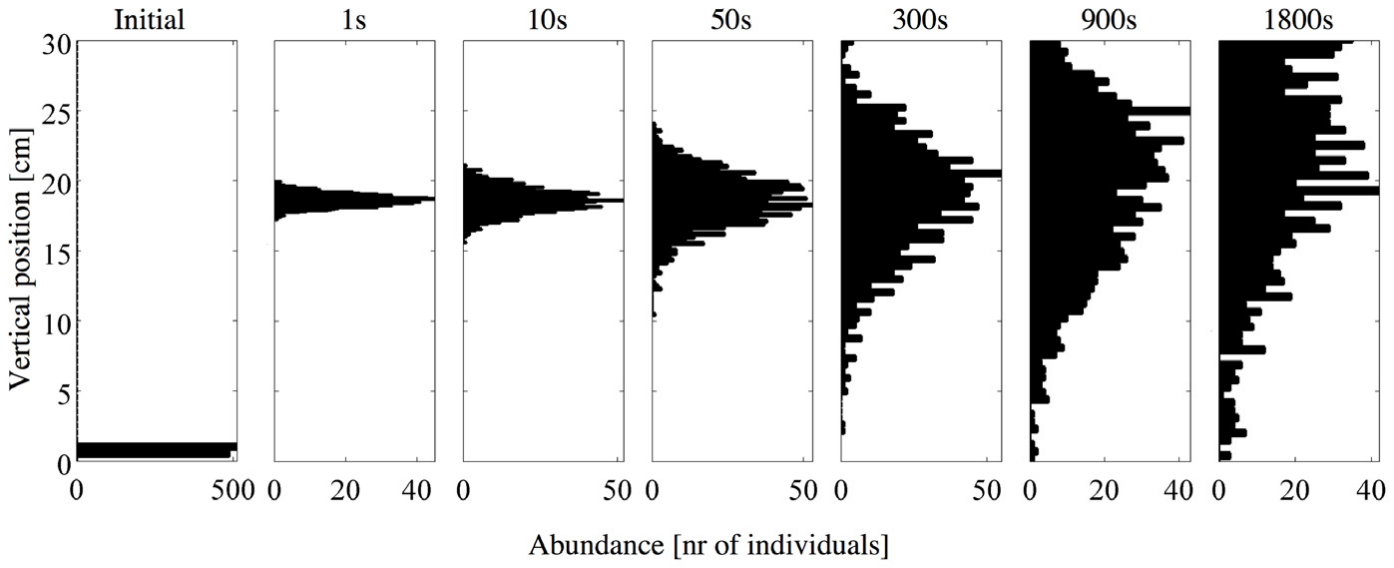
Individual-based model predictions of vertical population distributions of individuals performing a random walk with increasing correlation time, 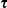 (stated in seconds above each panel). The initial distribution is shown in the left most panel. Duration of the simulation was 30 minutes for 1000 individuals. Note difference in x-axis ranges. Increases in 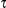 resulted in rapid increases in variance of the population distribution.

Predictions of population distribution from empirically measured vertical velocities through a series of individual-based simulation models showed that the empirically observed mean upward drift of the population was captured well by all model predictions irrespective of the assumed correlation time 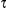 (Fig. 4). Correlation times of 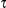 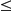
1 second predicted the population to tightly cluster vertically as cells moved upward through the water column (Fig. 4, panels 2 & 3). After 30 minutes of simulation, the mean vertical position of this population was 17.5 cm, with a standard deviation of 0.5 cm. The empirically observed, greater variance of the population dispersal and the delay in upward flux of the majority of the population were not predicted by model iterations assuming short correlation times. Simulations assuming minute-scale correlation times did capture the increased variance in population distribution (Fig. 4, panels 6 & 7).

**Figure 5:**
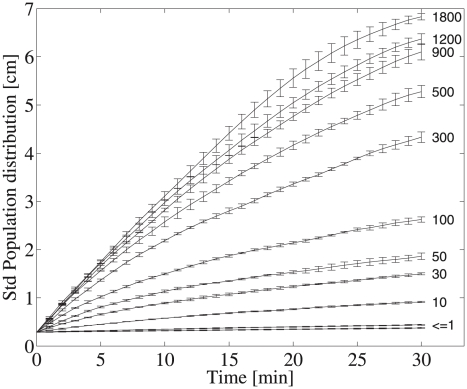
Mean standard deviations of model predicted population distribution over time as a function of 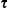. Errorbars show standard deviations of triplicate runs. Standard deviations were low and nearly constant for 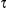 of 1 second or less. For correlation times 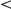
100 s the variability in population distribution increased moderately and rapidly for correlation times of 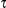 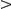
100 s. After 30 minutes, the standard deviation in population distribution for correlation times 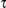 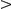
500 was over 20 fold greater than for an uncorrelated random walk.

Variance in population distribution increased rapidly with increasing correlation time over the first 30 minutes of model simulation (Fig. 5). Correlation times of 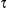 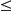
1 second resulted in low and near constant variance in population distributions. Increased correlation times of 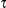 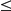
100 seconds lead to more rapid dispersal with standard deviations increasing by approximately 1 mm per minute. Assumed correlation times of 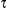 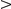
100 seconds predicted cells distributed throughout the water column and standard deviations of the population distributions increased at nearly 5 mm per minute. Increasing correlation time lead to emergence of the behavioral heterogeneity observed in the empirical data, signified by greater cell-to-cell variation in movement and resultant position.

**Figure 6:**
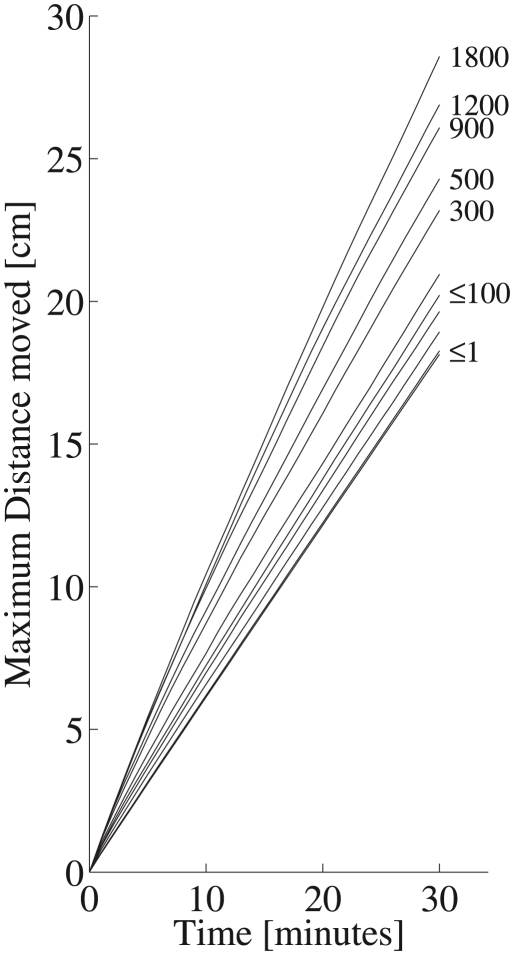
Mean distance farthest 25th percentile of the population moved in 30 minute simulations in an infinite water column. Maximum dispersal distance increased rapidly with increasing 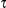 and model organisms with 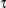 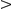
100 seconds were predicted to move on average two and up to three times farther than those with lower correlation times.

As is frequently observed (e.g. [9]), the uncorrelated random walk model predicted a gaussian cohort advancing upward at high cell concentration in close proximity. Longer correlation times resulted in rapid increases in population dispersal and more rapid spreading throughout the water column (Fig. 6). Although the *mean* net dispersal distance was identical, irrespective of the correlation time, long correlation times resulted in much higher variance in net dispersal distances because some individuals remained near the point of entry for the entirety of the model simulation, whereas few, fast upward swimming cells reached the surface of the tank within a few minutes. Behavioral heterogeneity was also suggested by the variance in the empirically measured vertical velocities (Fig. 1). Assumption of longer correlation times reproduced the observed cell-to-cell variation in motility, suggesting behavioral heterogeneity is an important contributor to the observed behaviors and population distributions, even though the source population was clonal.

**Figure 7:**
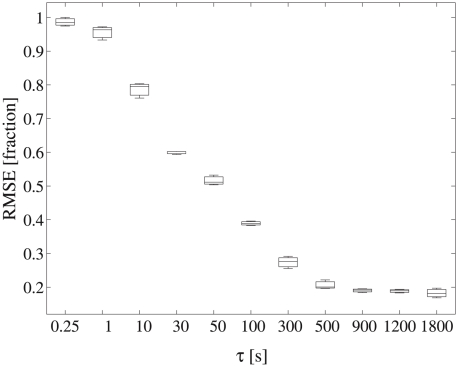
Root mean square error (RMSE) of triplicate, model predicted distributions decreased significantly with increasing correlation time 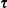 relative to the empirically measured distribution. Error bars are three standard deviations of the mean. RMSE was scaled to the maximum RMSE estimate at 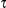 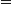
0.25 seconds. Empirical and simulation data were sampled with identical order and frequency. Model predictions for 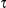 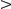
500 were statistically indistinguishable from one another and deviated the least from the empirical data.

Root mean square error (RMSE) of model predictions compared to the empirical distribution data decreased significantly with increasing correlation time 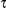 (Fig. 7). Model predictions differed most from empirical observations when assumed correlation was weak. Abundance predictions from highly correlated random walk models with 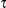 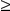
500 seconds differed least from the empirical data. RMSE was highest and statistically significantly different among models assuming 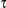 = 1 to 300 seconds. RMSE estimates for 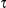 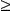
500 were lowest and statistically indistinguishable from one another, suggesting a minimum correlation time of 8 minutes. Further refinement or an upper limit of the correlation time was not identifiable based on this comparison of empirical and predicted population distributions.

The time and space scales of the model simulations were expanded to a 15 m water column and run for 24 hrs to explore the consequences of correlation duration on individual dispersal distances as well as population distributions. Total population size, evaluation frequency and all other aspects of the simulation were identical to those used in the simulations evaluated above and stated in the methods. Within patch retention mechanisms have been clearly demonstrated for this species [18] but were not implemented in the simulation.

First, expansion of the time and space scales of the model dimensions illustrated how longer correlation times increased population dispersal and thus variance in distribution. Based on the empirically measured, vertical velocity distribution, organisms moving with 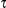 = 1 second occupied a vertical range of 10 cm after 12 hours. For organisms with correlation times of 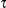 = 300 and 900 seconds, the predicted vertical ranges were 2.5 and 4 m respectively. Thus, correlation times increased population dispersal rates by at least an order of magnitude.

**Figure 8:**
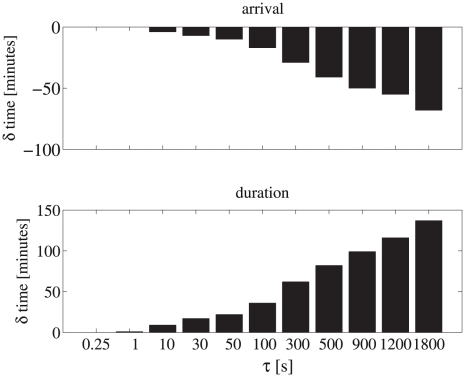
Relative time of first arrival (top panel) and residence duration (bottom panel) of the 25th fastest percentile of correlated random walkers in a simulated prey patch (1m thickness) within a 15 m water column. Difference in arrival and residence time were calculated relative to uncorrelated random walkers. Larger 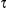 values result in over 1 hour earlier arrival and over 2 hour longer residence in the target prey patch.

Second, individual dispersal distance of the farthest traveling 25th percentile increased rapidly as correlation time increased. An individual with a correlation time of 900 seconds would travel on average twice as far and up to 3 times farther than an individual with a weakly correlated random walk. Therefore, individuals with highly correlated random walk behaviors are expected to encounter remote targets more rapidly than weakly correlated random walkers. Simulation of a 1m thick phytoplankton prey layer within the 15 m water column provides quantitative estimates of the impact of correlation time on the encounter of remote targets. Dimensions of the phytoplankton layer were based on empirical measurements in a shallow, coastal fjord [19]. Higher correlation times lead to considerably earlier arrival of 25% of the population within the prey layer, over 1 hour earlier in the case of 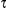 = 1800 seconds (Fig. 8, top panel). Populations with strongly correlated random walks remained within this prey layer over 2 hours longer than populations with weakly correlated random walks (Fig. 8, bottom panel).

## Discussion

Long correlation time and cell-to-cell variation were identified as key characteristics necessary to reproduce empirically observed population distributions from individual movement behaviors. Simultaneous measurements of both individual movement behaviors and population distributions were essential in linking microscopic movement behaviors with macroscopic population distributions. The results strongly suggest that motility patterns of some planktonic protists must have correlation times on the order of minutes, rather than seconds as is currently thought. Persistent similarity of movement in individual cells resulted in vastly higher dispersal rates for the population and significantly increased predicted rates of encounter with remote targets.

The correlation times estimated here far exceed previously measured correlation times. Previous studies suggest that transitions from highly correlated movements to more diffusive motion were observed to occur within 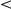
10 seconds for taxonomically diverse planktonic organisms ranging from bacteria to copepods [20], [21]. Uncorrelated movements were not identifiable in a set of several hundred movement tracks. To identify the correlation time-scales empirically would require minute-scale observations of 100s of individuals. The longer correlation times observed in this study may be due to the much larger than typical observation volume used, which may have resulted in longer free path lengths.

The consequences of long correlation times in individual motility patterns of plankton are significant. Planktonic organisms live in a nutritionally dilute environment (e.g. [?]). Recent, high-resolution observations in the ocean have shown that phytoplankton, the principal prey of many heterotrophic protists, are frequently concentrated in discrete layers or patches, rather than uniformly distributed [22]. Early hypotheses identified that planktonic predators must exploit these patches to sustain measured levels of secondary productivity [23]. Asexually reproducing organisms in particular can quickly transfer increased resource availability to increased growth and abundance. Long correlation times of individual movements result in significant increases in predicted dispersal distances of individuals and thus increases in the probable encounter with remote targets, including prey patches. Conversely, the probability of less advantageous encounters, including with predators is also increased [20] unless dispersive escape responses are evoked. For clonal organisms, increased probability of encountering unexploited resource patches may offset the increased risk of mortality due to predator encounters. The exact rate of encounter of remote targets will depend on the distribution, size and persistence of targets. Irrespective of target characteristics, individuals with long correlation time will encounter specific targets faster, given their, on average, almost two and up to three-fold greater dispersal distance. Behavioral modifications in response to prey derived stimuli that lead to consumer aggregations within resource patches are well documented (e.g. [18], [24], [25]) and are expected to provide further advantages to consumers exploiting dilute environments.

At the population level increased rates of population dispersal would erode aggregations and patchiness. It is noteworthy that the population also contained a small fraction of strong downward swimmers, which would further increase population dispersal rates. In the absence of aggregative stimuli the behavioral heterogeneity observed here may serve an important dispersive function and provide adaptive advantages to counteract long term aggregations. Long correlation times may have a homogenizing effect in light of many physical and biological processes that lead to cell aggregations and patchiness. This dispersive behavior could lead to reduced competition among cells [26], reduced risk from predators attracted to high cell concentrations [27] and reduced risk of the entire population being subjected to a localized risk or condition. Accelerated population dispersion may also counteract the tendency of cluster formation due to rapid asexual reproduction in planktonic organisms [28].

It is unknown how constant the measured rates of dispersal are over time. The observations made here were made shortly after organisms were introduced into the tank, thus population distributions were transient and dispersal rates likely at their maximum. The experimental set up deliberately did not include any stimulus that would either limit (e.g. aggregation) or enhance dispersal, so that measured dispersal rates were independent of external stimuli. However, organisms likely modulate their dispersal rates both over time and in response to external cues. Such modulation of correlation time has been suggested as an effective prey search strategy for organisms lacking sensory capacity [15]. Similarly, [29] have identified high variance in the turn rate of freshwater zooplankton (Daphnia spp.) and proposed that variation in movement behaviors has adaptive advantages.

The observed upward bias was a consistent characteristic of the measured swimming behaviors irrespective of the point of introduction or time of sampling. The same vertical bias was previously observed for the same species and the presence of a prey stimulus significantly reduced but did not eliminate upward bias [18]. In the absence of aggregative stimuli, this upward bias would ultimately lead to surface aggregations of organisms. Although surface aggregations were indeed observed in the laboratory, the stable, convection-suppressing conditions of this laboratory set up are neither realistic nor characteristic of planktonic habitats. The dynamic hydrography, including breaking internal waves, shear instability at boundaries and turbulent mixing, characteristic of the coastal ocean may counteract the observed net upward flux of organisms and prevent aggregations at the surface. Reported eddy diffusivities are an order of magnitude higher than the upward swimming velocities measured here and would counteract surface aggregations [30]. Given these dispersive factors, an inherent up swimming bias may hold adaptive advantages for planktonic organisms in the ocean, which is characterized by weak horizontal but strong and predictable vertical gradients in resource availability.

The data presented here strongly suggest that correlation times of motility patterns for some planktonic organisms are significantly longer than currently assumed. Long correlation times suggest that organisms with these motility patterns have higher dispersal rates and higher encounter rates with remote targets than organisms with only weakly correlated random walks. Simultaneous empirical observations of individual movement behaviors and the resulting population distributions were essential in linking statistical properties of cell movements to predictions of population distribution, a connection across disparate time and space scales. Model simulations of organism movements and population distributions were necessary to extrapolate beyond empirically measurable time and space scales. Verification of model predictions against empirical observations helped distinguish among a number of reasonable model formulations and ultimately in estimating the minimum correlation time. Quantifying the magnitude of the correlation time provides a basis for estimating individual encounter rates as well as population distributions. These quantitative tools are indispensable to predicting organism distributions and their function in the environment.

## Methods

### Culture of microorganisms

The heterotrophic dinoflagellate *Oxyrrhis marina* was used to study the effects of swimming behaviors on population dispersal. *O. marina* is 12–18 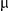
m in length and is a globally distributed species [31]. Cells swim and steer with the aid of perpendicular transverse and longitudinal flagellae that each propagate helicoidal waves [32]. *O. marina* was fed the haptophyte prey alga *Isochrysis galbana*, grown in nutrient-amended filtered seawater, f/2 [33]. Cultures were maintained on a 16∶8 hr light∶dark cycle, at 18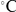 and 50 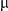
mol photons m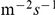 provided by cool and warm white lights. The cultures were not axenic. The salinity of the medium was 30. Both predator and prey cultures showed positive growth in all tested media ranging in salinity from 24 to 32. Cultures were transferred every 4–6 days to maintain exponential growth. Cell concentrations of both predator and prey cultures were determined with a Coulter Multisizer (Beckman Coulter, Miami, Florida) just prior to experiments. Predators were starved for 48 hrs prior to the experiment to minimize variation between cells.

### Empirical data collection & extraction

Organism swimming behaviors and vertical distributions were measured in complete darkness in a 1L, octagonal plexiglas tank of 30 cm height at ambient room temperature of approximately 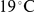. All organisms were introduced at the bottom of the tank and observations were made without external stimuli. To suppress water movement, the water column was stabilized through a weak, linear salinity gradient, ranging from 28 to 30. Video images were captured with two infra-red sensitive cameras (Cohu 4815-3000/000), equipped with Nikon 60 mm Micro Nikkor lenses and illuminated by infra-red light emitting diodes (Ramsey Electronics, 960 nm). The cameras were mounted on a vertically movable stage. Vertical position was controlled through a ruler fixed to the side of the stage. Video was recorded at 15 frames per second. Prior to these experiments, it was verified that some cells reached the top of the experimental tank within 15 minutes and filming was commenced after a waiting period of 15 minutes. Footage was collected in the center of the water column at six equally spaced horizons, approximately 5 cm apart, for 2 minutes every 20 to 30 minutes for a total duration of 1.5 hours. This resulted in 3 video segments being collected at each horizon. At the beginning of the experiment the order of sampling horizons was randomized. The position of organisms in the video footage was determined with ImageJ image processing software by removing stationary background objects and thresholding. A three-dimensional calibration grid was used to convert video pixel dimensions to physical units. The stereoscopic field of view was approximately 1.8 cm wide, 1.3 cm high and 4.0 cm deep. Thus, cells within a volume of approximately 9 ml were observed. These movement data were unencumbered by frequently encountered methodological limitations such as low temporal resolution, physical restriction (e.g. container size) and the 3D rendition avoids underestimates of swimming velocities and directions. Three-dimensional swimming paths were generated from pixel positions by Tracker*3D*, a Matlab-based motion analysis package for tracking organism movement written by Danny Grünbaum (Univ. of Washington). Before analysis, swimming paths were smoothed with a cubic spline to remove high-frequency noise. Individual movement statistics were calculated from 3D swimming paths, subsampled at 0.25 second intervals, including only trajectories of at least 3 seconds duration. Abundance of *O. marina* was estimated from the average number of 3D trajectories observed in each video frame. Further details on the water column set-up, filming and data collection are reported in [18].

### Model formulation

An individual-based, hidden Markov model was formulated to predict the vertical redistribution of the *Oxyrrhis marina* population in the water column. The successive positions and movement parameters for each individual were modeled explicitly based on the empirically observed behaviors. The magnitude and frequency distribution of empirically measured vertical velocities provided the basis for modeled velocities (Fig. 1). Negative velocities indicate downward movement. The 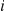-th model organism at time 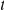 was characterized by a position 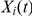, vertical velocity 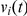 and associated with a specific swimming trajectory 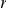, randomly drawn from the entirety of observed paths and then assigned the first velocity measured within that path. Triplicate model iterations were evaluated at time increments of 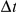 4 Hz with 1000 individuals each. Successive organism positions were calculated as:
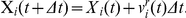
Model organisms encountering the upper or lower boundaries were assigned movement paths with net downward or upward movements respectively. The model was chosen to be 1-dimensional, since there were no horizontal gradients in external stimuli and the variable of interest was the rate of vertical population redistribution in the water column. The spatial and temporal scales of the model were identical to the laboratory set up.

### Implementation of correlation time

In all model formulations, individuals were randomly assigned new velocities at the model iteration frequency of 4 Hz. In the uncorrelated random walk model, the assigned vertical velocity was drawn from the entirety of all observed vertical velocities. Thus, information on the associated trajectory 
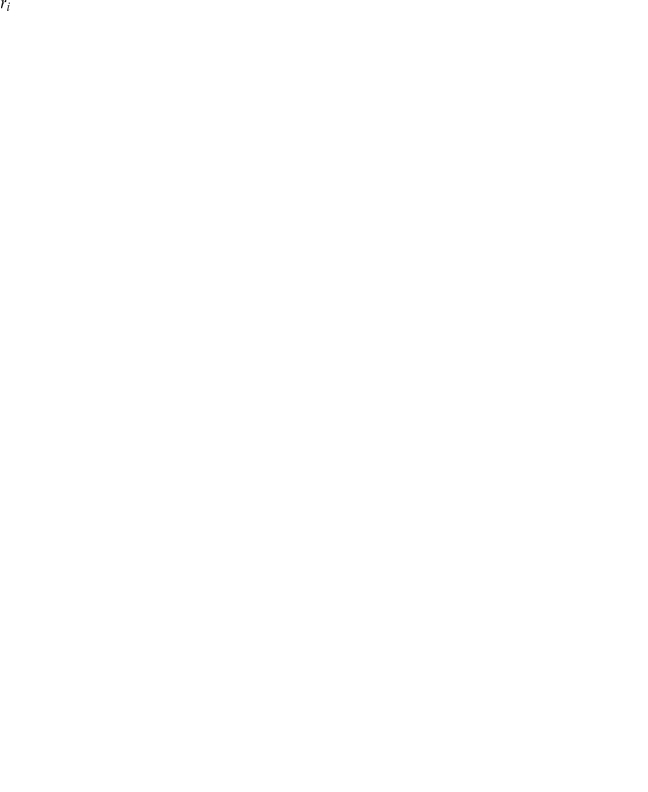
 was meaningless for the uncorrelated random walk model. In the correlated random walk model, subsequent velocities were sampled from the associated swimming trajectory 
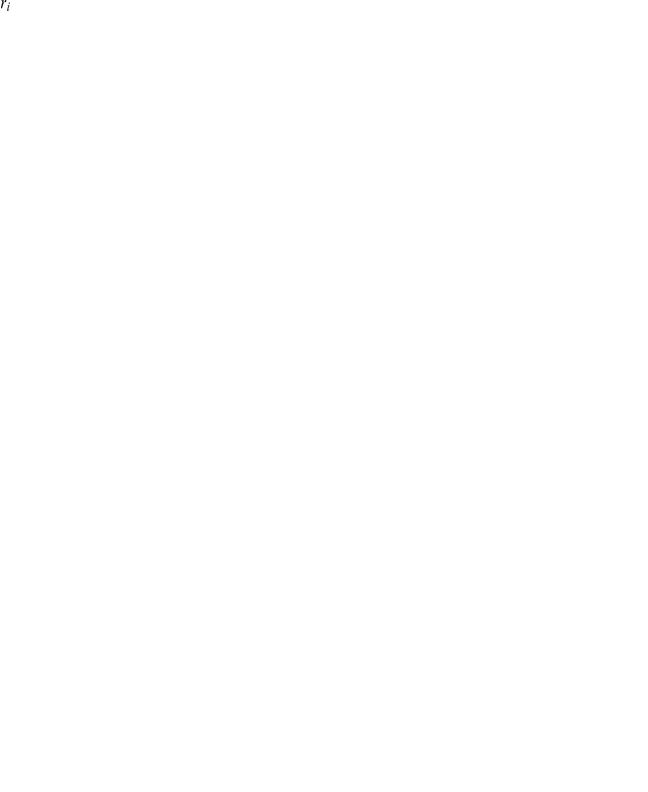
 in sequence of observation. New trajectories were assigned at the frequency 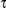 with probability
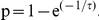
Thus, at 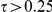 seconds (i.e. the iteration frequency of 4 Hz), individuals sampled repeatedly and in sequence from the velocities within one empirically determined trajectory. Modeled correlation time 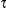 ranged from 0 to 1800 seconds. Population size was held constant, since demographic processes were not expected to change abundance over the model duration of 1.5 hrs.

### Statistical analysis

Comparisons of the vertical velocities over time and at different filming horizons were made using a two-way ANOVA. Sensitivity analyses were conducted to ensure that the path discretization parameters did not significantly change the calculated vertical velocities. Furthermore, artificial data sets were created to test the sensitivity of model predictions to deviations from normality for the frequency distribution of vertical velocities and total sample size. Neither analysis suggested a change in conclusions. The autocorrelation coefficients of vertical velocities were calculated for each path separately, with mean velocity subtracted. To facilitate comparison among paths, correlation coefficients were normalized. The root mean square error (RMSE) between empirically observed and model predicted vertical population distributions were calculated to facilitate among model comparisons. To do so, vertical population distribution from model predictions were sampled in the same order and frequency as the empirical data were collected. All RMSE estimates were scaled to the maximum RMSE observed to remove the effect of sample size from estimates. Comparison of RMSE were made with a one-way ANOVA. Significant differences among means were assessed using a Bonferroni corrected post-hoc test. Statistical significance was assigned at 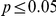. All analyses and simulations were done using the software package Matlab 7.9.0..
